# JMJD2B is required for *Helicobacter pylori*-induced gastric carcinogenesis via regulating COX-2 expression

**DOI:** 10.18632/oncotarget.9573

**Published:** 2016-05-24

**Authors:** Fengjuan Han, Juchao Ren, Jinjin Zhang, Yundong Sun, Fang Ma, Zhifang Liu, Han Yu, Jihui Jia, Wenjuan Li

**Affiliations:** ^1^ Department of Microbiology, Key Laboratory for Experimental Teratology of Chinese Ministry of Education, School of Medicine, Shandong University, Jinan, PR China; ^2^ Department of Biochemistry and Molecular Biology, School of Medicine, Shandong University, Jinan, PR China; ^3^ Department of Urology, Qilu Hospital, Shandong University, Jinan, PR China

**Keywords:** JMJD2B, KDM4B, Helicobacter pylori, COX-2, gastric cancer

## Abstract

Helicobacter pylori (*H. pylori*) infection is the strongest risk factor for the initiation and progression of gastric cancer. However, the mechanism of *H. pylori*-induced pathogenesis remains unclear. In this study, we investigate the role of *H. pylori* infection in JMJD2B upregulation and the mechanism underlying gastric carcinogenesis. We find that JMJD2B can be induced by *H. pylori* infection via β-catenin pathway. β-catenin directly binds to JMJD2B promoter and stimulates JMJD2B expression following *H. pylori* infection. Increased JMJD2B, together with NF-κB, binds to COX-2 promoter to enhance its transcription by demethylating H3K9me3 locally. JMJD2B and COX-2 expression is upregulated in *H. pylori* infected mice *in vivo*. Furthermore, JMJD2B and COX-2 expression is gradually increased in human gastric tissues from gastritis to gastric cancer. The level of JMJD2B and COX-2 in *H. pylori*-positive gastritis tissues is significantly higher than that in *H. pylori*-negative tissues. Moreover, a positive correlation between JMJD2B and COX-2 expression is found in both gastritis and gastric cancer tissues. Therefore, JMJD2B is a crucial factor in triggering *H. pylori*-induced chronic inflammation and progression of gastric carcinogenesis and it may serve as a novel target for the intervention of gastric cancer.

## INTRODUCTION

Gastric cancer is the fifth most common malignancies and the third leading cause of cancer-related death among all cancers worldwide. According to the global cancer statistics in 2012, an estimated 951,600 new stomach cancer cases and 723,100 deaths occurred [1]. Since it is lack of reliable early diagnostic markers, most patients are diagnosed at the advanced or metastatic stage. Surgery, chemotherapy or radiotherapy plays a minor role to improve the survival rate [2]. Therefore, it is urgent to identify new early diagnostic markers and explore novel therapeutic targets.

*H. pylori* infection and the resulting gastric inflammation is the strongest identified risk factor for the development of gastric cancer [3]. Persistent infection with *H. pylori* may cause chronic atrophic gastritis, with development of intestinal metaplasia, dysplasia, and gastric carcinoma [4], and *H. pylori* has been classified as a class I carcinogenic factor by the International Agency for Research on Cancer since 1994. Recent studies have reported that *H. pylori*-induced epigenetic modifications, such as DNA methylation and histone modifications, are critical in many oncogenic processes including regulating gene expression, cell cycle and cell proliferation [5, 6]. Dysregulated epigenetic alterations, especially in early neoplastic development, may be just as significant as genetic mutations in driving cancer development and progression [7]. However, the role of *H. pylori* infection in these processes has not been well defined. JMJD2B, also known as KDM4B, is newly identified and characterized as a member of the histone demethylase JMJD2 family. JMJD2B specifically targets the trimethylated lysine 9 of histone H3 (H3K9) in pericentric heterochromatin and euchromatin [8–10], which has been implicated in inflammation [11], and tumorigenesis [12]. In our previous work, JMJD2B is overexpressed in gastric cancer and plays a role in tumor cell proliferation, survival, invasion and metastasis [13, 14]. However, the precise mechanism of JMJD2B upregulation in gastric cancer has not been completely elucidated. Given the role of *H. pylori* infection in gastric malignant transformation, we investigate the link between *H. pylori* infection and JMJD2B expression associated with gastric cancer pathogenesis.

Recent studies suggest that COX-2 is activated in *H. pylori*-associated gastritis and gastric cancer [15]. COX-2 is reported to be significantly correlated with angiogenesis [16], inhibition of apoptosis [17] and invasiveness [18] in gastric cancer. Although the oncogenic mechanisms of COX-2 and *H. pylori*-induced inflammation in gastric carcinogenesis have been illuminated, the precise molecular mechanism of its activation is not yet completely defined. Accumulating evidence suggest that COX-2 expression is regulated by epigenetic mechanisms, which associated with the clinical outcome of gastric cancer [19, 20]. Therefore, it arouses our interest to see whether *H. pylori*-induced COX-2 upregulation may be controlled by JMJD2B-mediated epigenetic mechanisms.

In the present work, we investigated the role of JMJD2B in *H. pylori*-induced gastric carcinogenesis and discovered a novel intracellular signaling pathway involving JMJD2B and COX-2 in *H. pylori*-mediated pathogenesis.

## RESULTS

### Induction of JMJD2B expression by *H. pylori* in gastric epithelial cells

**Figure 1:**
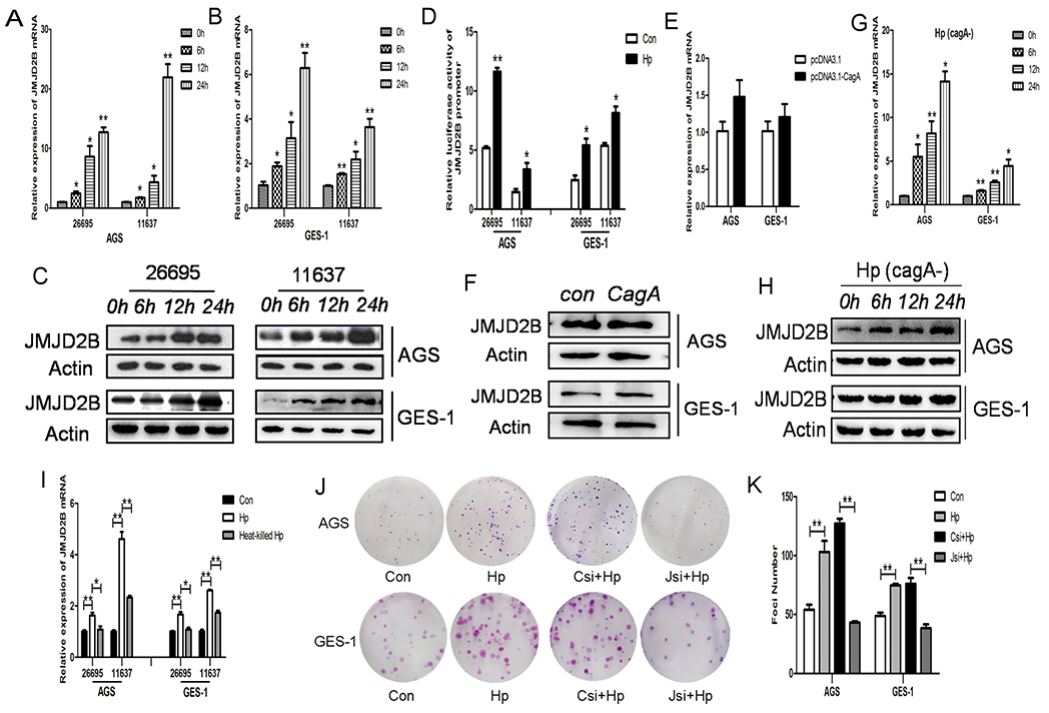
JMJD2B is induced by *H. pylori* infection independent of CagA Infection experiments were conducted with MOI (multiplicity of infection) of 100:1. **A-C.** qRT-PCR and Western blot analysis of JMJD2B mRNA and protein levels in AGS and GES-1 cell lines infected with *H. pylori* at indicated time points. **D.** Luciferase activity of JMJD2B promoter in both cells with *H. pylori* infection for 12h. **E-F.** qRT-PCR and Western blot analysis of mRNA and protein levels of JMJD2B in AGS and GES-1 cell lines transfected with control and CagA plasmid. **G-H.** qRT-PCR and Western blot analysis of JMJD2B expression in both cell lines infected with CagA-depleted *H. pylori*. **I.** qRT-PCR analysis of JMJD2B mRNA level in *H. pylori* or heat-killed *H. pylori*-infected AGS and GES-1 cells. **J-K.** The colony formation assay was assessed in control cells or JMJD2B knockdown cells with *H. pylori* infection for 12h. The number of colonies was analyzed. *P <0.05, **P <0.01 compared with control. Data are mean ± SEM of 3 independent experiments.

First, immortalized human gastric epithelial cells (GES-1) and poorly differentiated gastric cancer cells (AGS) were used to study the relationship between *H. pylori* infection and JMJD2B expression. JMJD2B mRNA and protein levels were significantly increased over time with *H. pylori* infection in both cell lines (Figure 1A-1C). Similarly, the luciferase assay showed that JMJD2B promoter activity was stimulated by *H. pylori* infection in both cells (Figure 1D). These findings demonstrated that *H. pylori* induced JMJD2B upregulation at transcriptional level. To further determine which factor of *H. pylori* was responsible for the increased JMJD2B expression, CagA plasmid, CagA-depleted *H. pylori* strain and heat-inactivated *H. pylori* were used. qRT-PCR and western blot analysis showed that CagA did not promote the mRNA and protein levels of JMJD2B in AGS and GES-1 cells (Figure 1E-1F). Furthermore, when cells were incubated with CagA-depleted *H. pylori* strain, a comparable increase in JMJD2B expression was detected (Figure 1G-1H). Thus, *H. pylori* CagA is not the factor that stimulates JMJD2B expression. The mRNA level of JMJD2B was not increased in AGS and GES-1 cells after heat-inactivated *H. pylori* infection (Figure 1I), suggesting that physical interaction of viable bacteria was required to induce JMJD2B expression. We further examined the biological function of JMJD2B on *H. pylori*-induced clonogenic potential in both cells. As shown in Figure 1J-1K, *H. pylori* infection promoted clonogenic ability of both cells, and this promotion was restrained by knockdown of JMJD2B.

### β-catenin stimulates *H. pylori*-induced JMJD2B upregulation

**Figure 2:**
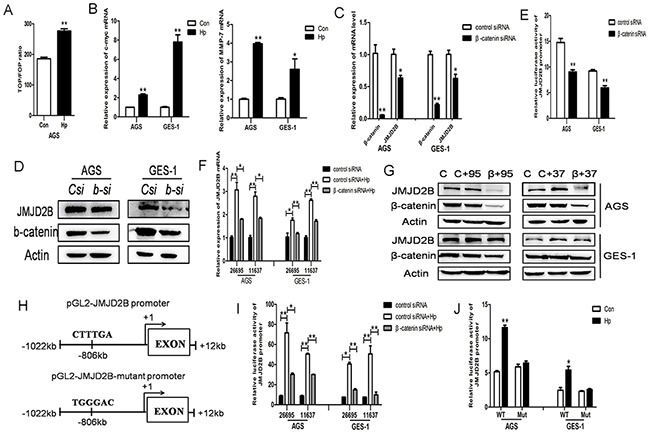
β-catenin stimulates *H. pylori*-induced JMJD2B upregulation **A.** AGS cells were transiently transfected with pTOP-FLASH or pFOP-FLASH reporter plasmids separately and cotransfected with TK plasmid. 48h later, the cells were infected with *H. pylori* for 12h and luciferase activity was examined. **B.** qRT-PCR analysis of MMP-7 and c-myc, the target genes of β-catenin, was performed after *H. pylori* infection in AGS and GES-1 cells. **C-D.** qRT-PCR and western blot analysis of mRNA and protein levels of β-catenin and JMJD2B in control and β-catenin siRNA-transfected AGS and GES-1 cells. **E.** Luciferase activity of JMJD2B promoter in control and β-catenin siRNA-transfected AGS and GES-1 cells. **F-G.** qRT-PCR and Western blot analysis revealed that knocking down β-catenin expression by siRNA suppressed *H. pylori*-induced upregulation of JMJD2B mRNA and protein levels. **H.** Schematic structure of pGL2-JMJD2B promoter and pGL2-JMJD2B mutant promoter. **I.** Luciferase reporter assay revealed that *H. pylori*-induced JMJD2B promoter activity was significantly inhibited by β-catenin knockdown. **J.** Luciferase activity was determined with the wild-type or mutant JMJD2B reporter transfection after *H. pylori* infection. * *P* <0.05, ** *P* <0.01 compared with control. Data are mean ± SEM of 3 independent experiments.

Since β-catenin pathway is involved in *H. pylori*-induced inflammatory response and gastric tumorigenesis, we determined the effect of *H. pylori* infection on β-catenin activation in our system. Luciferase reporter assay showed significantly increased levels of TOP/FOP ratio compared to control in AGS cells (Figure 2A). The upregulation of β-catenin target genes MMP-7 and c-myc were also detected after *H. pylori* infection (Figure 2B). The above results demonstrated that *H. pylori* induced β-catenin activation in our cell lines. Analysis of JMJD2B promoter sequence showed a potential binding site of TCF/LEF and our previous study had shown a physical interaction of JMJD2B and β-catenin in gastric cancer cells, we then asked whether β-catenin regulated JMJD2B expression in these cells. JMJD2B mRNA and protein levels were significantly decreased in AGS and GES-1 cells after knocking down β-catenin expression using specific siRNA (Figure 2C-2D). The luciferase assay revealed that inhibition of β-catenin expression reduced the luciferase activity driven by the JMJD2B promoter (Figure 2E). Finally, we examined the role of β-catenin in *H. pylori*-induced JMJD2B expression. It can be seen from Figure 2F-2G that β-catenin silencing inhibited *H. pylori*-induced JMJD2B upregulation. To further determine the regulatory mechanism, we constructed pGL2-JMJD2B promoter vector containing the TCF/LEF binding site CTTTGA which can be bound by β-catenin and pGL2-JMJD2B-mutant promoter vector where β-catenin binding site was mutated to TGGGAC (Figure 2H). The luciferase assay showed that *H. pylori*-induced JMJD2B promoter activity was dramatically attenuated by knocking down β-catenin (Figure 2I). JMJD2B promoter activity was not increased after mutation of the putative β-catenin binding site, which showed that β-catenin was required for the transcription activation of JMJD2B following *H. pylori* infection (Figure 2J). Taken together, these results reveal that *H. pylori* activates β-catenin activity increasing JMJD2B transcription in gastric epithelial cells.

### JMJD2B is required for *H. pylori*-induced COX-2 activation

**Figure 3:**
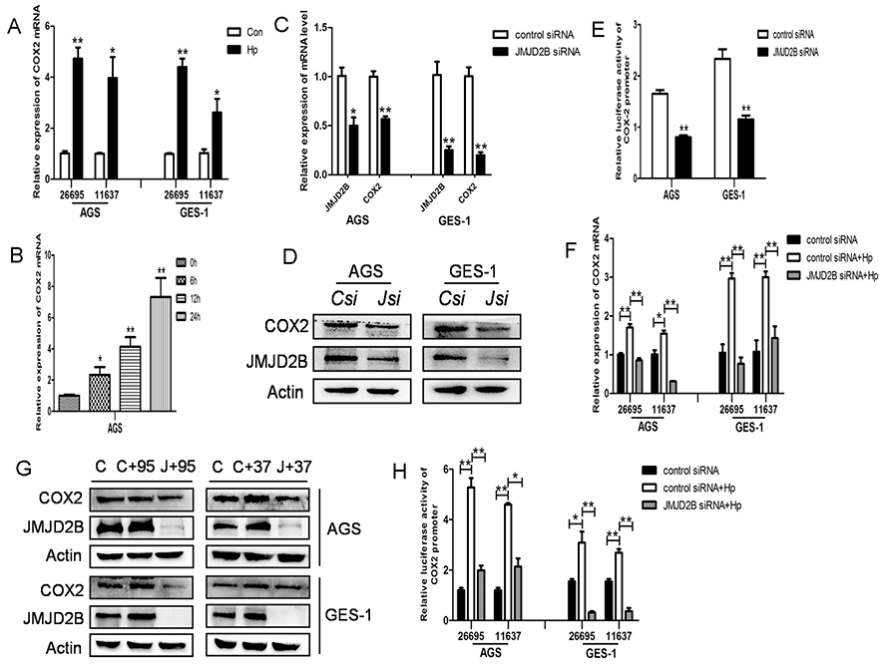
*H. pylori*-induced COX-2 activation is JMJD2B-dependent **A.** qRT-PCR analysis of COX-2 mRNA level in *H. pylori*-infected AGS and GES-1 cells. **B.** qRT-PCR analysis of COX-2 mRNA level in CagA-depleted *H. pylori*-infected AGS cells. **C-D.** qRT-PCR and Western blot analysis of mRNA and protein levels of JMJD2B and COX-2 in JMJD2B siRNA-transfected AGS and GES-1 cells. **E.** Luciferase activity of COX-2 promoter in AGS and GES-1 cells by JMJD2B knocking down. **F-G.** qRT-PCR and Western blot analysis showed that inhibition of JMJD2B by siRNA suppressed *H. pylori*-induced COX-2 mRNA and protein levels. **H.** Luciferase reporter assay showed that *H. pylori*-induced COX-2 promoter activity was inhibited by JMJD2B knockdown. * *P* <0.05, ** *P* <0.01 compared with control. Data are mean ± SEM of 3 independent experiments.

Recent studies have reported the induction of COX-2 by *H. pylori* infection. Here, we also found that COX-2 mRNA level was elevated in GES-1 and AGS cells after *H. pylori* infection (Figure 3A). Besides, *H. pylori* activated COX-2 expression independent of CagA (Figure 3B). Since JMJD2B was also increased by *H. pylori* in a CagA-independent manner, we assessed the role of JMJD2B in *H. pylori*-induced COX-2 upregulation. As shown in Figure 3C-3D, COX-2 mRNA and protein levels were both decreased by JMJD2B knockdown compared with control in AGS and GES-1 cells. Luciferase reporter assay showed that inhibition of JMJD2B significantly reduced the luciferase activity driven by the COX-2 promoter (Figure 3E). These results suggested that JMJD2B induced COX-2 transcriptional activation in gastric epithelial cells. To further confirm *H. pylori*-induced COX-2 expression is regulated by JMJD2B, GES-1 and AGS cells were transfected with JMJD2B siRNA and then infected with *H. pylori*. *H. pylori*-induced increase in COX-2 mRNA and protein levels were blocked by JMJD2B silencing (Figure 3F-3G). Similarly, *H. pylori*-induced COX-2 promoter activity was significantly inhibited by JMJD2B knockdown (Figure 3H). Therefore, these findings indicate that JMJD2B is required for *H. pylori*-induced COX-2 expression.

### JMJD2B regulates COX-2 expression via cooperating with NF-κB in gastric epithelial cells

**Figure 4:**
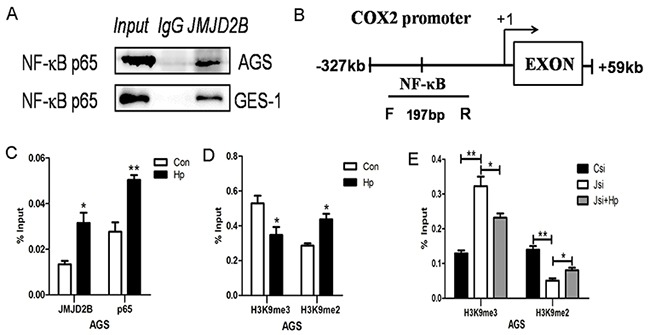
JMJD2B cooperates with NF-κB to regulate COX-2 expression in gastric epithelial cells **A.** Interaction between JMJD2B and p65. AGS and GES-1cells infected with *H. pylori* were immunoprecipitated with anti-JMJD2B antibody and the coimmunoprecipitated p65 was detected using anti-p65 antibody. **B.** Schematic representation of COX-2 promoter showing the locations of the PCR primer used for ChIP assay. **C-D.** ChIP-qRT-PCR was performed in AGS cells to evaluate the enrichment of JMJD2B, NF-κB and histone H3K9me3 and H3K9me2 on the COX-2 promoter region. **E.** The methylation level of H3K9 in the COX-2 promoter region under the presence and absence of JMJD2B as well as *H. pylori* infection. * *P* <0.05, ** *P* <0.01 compared with control. Data are mean ± SEM of 3 independent experiments.

Next, we determined the molecular mechanism that JMJD2B regulated COX-2 expression. As a histone demethylase, JMJD2B usually cooperates with transcription factors to regulate gene expression by demethylating histone H3K9me3. NF-κB has been demonstrated to be a major transcription factor regulating COX-2 expression, we asked whether JMJD2B modulated COX-2 expression by interaction with NF-κB during *H. pylori* infection. To this end, we performed Co-IP assay in AGS and GES-1 cells after *H. pylori* infection and a physical interaction of JMJD2B and NF-κB was detected (Figure 4A). Then, ChIP assay was prepared after *H. pylori* infection and ChIP-qRT-PCR was performed using specific primers amplifying the COX-2 promoter region (from −327 to +19) containing a NF-κB binding site (Figure 4B). We found that *H. pylori* significantly increased the binding of JMJD2B and NF-κB (p65) to COX-2 promoter region in AGS cells (Figure 4C). Moreover, a marked reduction of H3K9me3 and sharp accumulation of H3K9me2 at the COX-2 promoter was observed (Figure 4D). Finally, we further investigated the H3K9 methylation pattern in the COX-2 promoter in the presence and absence of JMJD2B as well as in the presence and absence of *H. pylori* infection. JMJD2B depletion significantly enhanced H3K9me3 level at the COX-2 promoter region, but the increase of H3K9me3 was inhibited by *H. pylori* infection (Figure 4E). Moreover, a reduction of H3K9me2 was observed after JMJD2B knockdown, while the reduction was restored by *H. pylori* infection (Figure 4E). Therefore, *H. pylori* increases binding of JMJD2B to COX-2 promoter where it demethylates H3K9me3 and recruits NF-κB to bind on COX-2 promoter to facilitate COX-2 induction.

### *H. pylori* regulates JMJD2B and COX-2 expression *in vivo*

**Figure 5:**
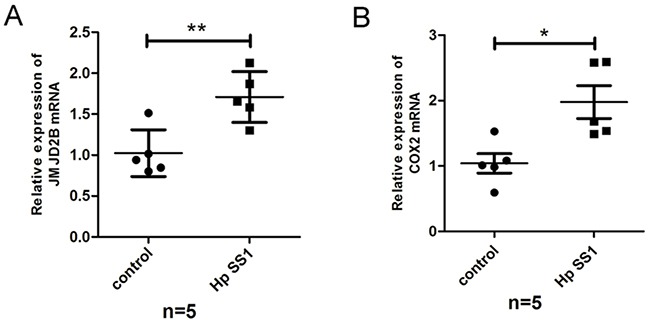
*H. pylori* regulates JMJD2B and COX-2 expression *in vivo* **A-B.** qRT-PCR analysis of mRNA levels of JMJD2B and COX-2 in gastric mucosal samples of *H. pylori*-infected mice. * *P* <0.05, ** *P* <0.01.

To investigate whether *H. pylori* induces JMJD2B and COX-2 activation *in vivo*, C57BL/6J mice were infected with *H. pylori* (SS1) or PBS using intragastric administration and killed at 8wk after inoculation. qRT-PCR analysis showed that the mRNA levels of JMJD2B and COX-2 were both increased in *H. pylori*-infected gastric mucosal samples (Figure 5A-5B).

### JMJD2B is overexpressed in human gastritis and gastric cancer tissues

**Figure 6:**
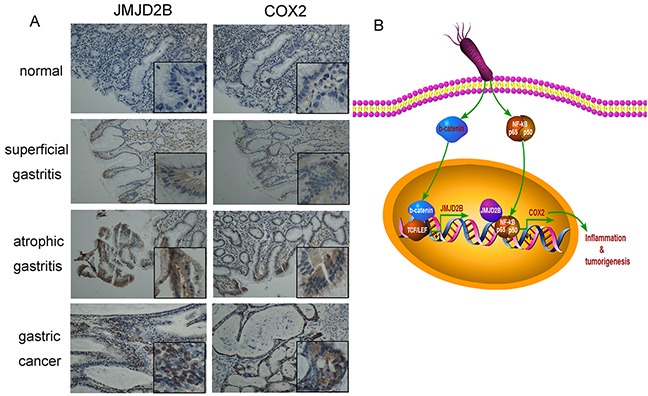
Expression of JMJD2B and COX-2 in human clinical samples from chronic inflammation to gastric cancer **A.** Expression of JMJD2B and COX-2 is examined by immunohistochemistry staining in normal, superficial gastritis, atrophic gastritis and gastric cancer samples. Representative images are shown. Original magnification: ×200, magnification in the box: ×400. **B.** An outline of the main pathways illustrated in our work.

Finally, we determined whether the results from the cell lines and animal experiments have any clinical relevance. Immunohistochemical staining showed that JMJD2B was gradually increased in human specimens from gastritis to gastric cancer (Figure 6A). Moreover, the positive staining of JMJD2B were detected in *H. pylori* positive gastritis samples (61.5%, 8/13), which was higher than that of *H. pylori* negative samples (15.4%, 6/39) (Table 1). We also found that JMJD2B expressed in 20% (7/35) superficial gastritis tissues and in 66.7% (8/12) atrophic gastritis tissues. These results indicate that JMJD2B is involved in the process of *H. pylori*-induced inflammation to gastric cancer.

**Table 1:** Association between JMJD2B expression and *H. pylori* infection in chronic gastritis patients

Variables	Total	JMJD2B expression		P value
		0-1	2-3	
*H. pylori*				
Positive	13	5	8	P<0.01
Negative	39	33	6	

NOTE: 0-1, low expression of genes; 2-3, high expression of genes.

χ^2^ test

### JMJD2B expression positively correlates with COX-2 in chronic gastritis and gastric cancer tissues

We also detected COX-2 expression in gastritis and gastric cancer tissues. COX-2 level was consistent with the expression trends of JMJD2B in gastritis and gastric cancer samples (Figure 6A). Moreover, a strong positive correlation between JMJD2B and COX-2 levels in gastritis (Table 2) and gastric cancer (Table 3) tissues was found.

**Table 2:** Association between expression of JMJD2B and COX-2 in human gastritis mucosa

Variables	Total	JMJD2B expression		P value
		0-1	2-3	
COX-2 expression				
0-1	38	32	6	P<0.01
2-3	14	3	11	

NOTE: 0-1, low expression of genes; 2-3, high expression of genes.

χ^2^ test

**Table 3:** Association between expression of JMJD2B and COX-2 in human gastric cancer tissues

Variables	Total	JMJD2B expression		P value
		0-1	2-3	
COX-2 expression				
0-1	18	14	4	P<0.05
2-3	14	6	8	

NOTE: 0-1, low expression of genes; 2-3, high expression of genes.

χ^2^ test

## DISCUSSION

Although numerous studies have been conducted in *H. pylori* pathogenesis and carcinogenesis, the detailed mechanism remains undefined. In this study, we focus on the critical role of histone demethylase JMJD2B in the transformation from chronic inflammation to gastric cancer induced by *H. pylori*. We demonstrate that JMJD2B is induced by *H. pylori* in a CagA-independent manner in both gastric epithelial cells and gastric cancer cells. Knockdown of JMJD2B significantly inhibits *H. pylori*-induced cell proliferation *in vitro*. *H. pylori* infection induces β-catenin nuclear translocation and lymphocyte enhancer factor/T cell factor (LEF/TCF) transactivation. β-catenin, together with LEF/TCF binds to JMJD2B promoter and activates its expression. The upregulated JMJD2B then stimulates COX-2 transcription by cooperating with NF-κB, which eventually promotes *H. pylori*-induced carcinogenic process.

It is well known that *H. pylori*-induced chronic inflammation is one of the leading factors for the initiation and development of gastric carcinoma [3]. Numbers of studies have been done to elicit the association between *H. pylori* infection and gastric cancer. *H. pylori* activates multiple intracellular pathways in epithelial cells, such as NF-κB, β-catenin pathway and PI3K/AKT pathway. These pathways affect various cellular functions, leading to increased inflammatory cytokine production, altered apoptosis rate, deregulated epithelial cell proliferation and differentiation, and finally resulting in gastric oncogenic transformation [21–24]. Moreover, *H. pylori*-induced epigenetic alterations, especially histone modifications, have been strongly implicated in gene-expression alteration and tumorigenesis [25, 26]. Infection with *H. pylori* causes acetylation of H4, which is associated with p21 gene expression in gastric epithelial cells [27]. *H. pylori* induces *cag* pathogenicity island-dependent H3S10 phosphorylation, which is associated with *c-Jun* upregulation and *hsp70* downregulation and cell cycle premitotic arrest [6]. As an epigenetic molecule, JMJD2B has been reported to possess oncogenic activities in human cancers [12, 28]. Recently, we demonstrated that JMJD2B promoted cell proliferation, survival, invasion and metastasis in gastric cancer [13, 14], indicating that JMJD2B plays a key role in gastric tumorigenesis and progression. However, little is known about the involvement of JMJD2B in *H. pylori*-induced inflammation to cancer.

In this work, we investigated the early transcription of JMJD2B after *H. pylori* infection. Both the mRNA and protein levels of JMJD2B were significantly increased in response to *H. pylori* infection in different gastric epithelial cells, and the induction was not dependent on the CagA status. We also determined that JMJD2B stimulated cell clonogenic ability induced by *H. pylori*. These results indicate that JMJD2B activation may be an important step in *H. pylori* pathogenesis.

Among the signaling pathways *H. pylori* activated, β-catenin plays an important role in inflammation and gastric carcinogenesis. [29]. *H. pylori* infection induces β-catenin transcriptional activity, LEF/TCF activation, and target genes expression, which are involved in many cellular processes including cell cycle control, differentiation and cell migration [30, 31]. We previously reported that JMJD2B was physically associated with β-catenin and enhanced its nuclear localization and transcription [14], then we determined the role of β-catenin in the regulation of JMJD2B induced by *H. pylori*. As expected, we found a potential binding site of β-catenin on JMJD2B promoter. Furthermore, our results revealed that β-catenin upregulated JMJD2B expression by increasing JMJD2B promoter transcription activity following *H. pylori* infection.

Since COX-2 is known to be associated with *H. pylori*-induced inflammatory response and tumorigenesis [32, 33], illuminating the mechanism that how COX-2 is deregulated would help shed new light on *H. pylori* pathogenicity. Here we determined that JMJD2B upregulated *H. pylori*-induced COX-2 transcription. It has been reported that *H. pylori* induces COX-2 expression by activating transcription factor NF-κB, which is a key regulator of immune and inflammatory responses and regulates many processes in carcinogenesis [34]. Our Co-IP and ChIP assays showed the interaction between JMJD2B and NF-κB in gastric cancer cells and the increased binding of JMJD2B with NF-κB to COX-2 promoter coupled with the decrease of H3K9me3 and increase of H3K9me2 by *H. pylori* infection.

Consistent with the results from cell lines, JMJD2B and COX-2 expression was increased in *H. pylori*-infected gastric tissues *in vivo*. Furthermore, overexpression of JMJD2B and COX-2 was observed in clinical specimens from gastritis to gastric cancer and JMJD2B level was significantly associated with *H. pylori* infection (P<0.01). We also found that JMJD2B positively correlated with COX-2 expression in both gastritis and gastric cancer tissues. These findings indicate that JMJD2B activation and COX-2 upregulation contribute to gastric inflammation and carcinogenesis.

In conclusion, we determined the role and regulatory mechanism of JMJD2B in the progress of gastric inflammation to gastric cancer induced by *H. pylori* infection. The *H. pylori*-β-catenin-JMJD2B-COX-2 signaling cascade may be a new mechanism for the initiation and development of gastric cancer. JMJD2B may serve as a novel target for the treatment and early intervention of gastric cancer.

## MATERIALS AND METHODS

### Cell culture, siRNA and plasmid transfection

Human gastric epithelial cell line GES-1 and human gastric cancer cell line AGS were purchased from the Cell Resource Center, Shanghai Institute of Biochemistry and Cell Biology at the Chinese Academy of Sciences (Shanghai, PR China). GES-1 and AGS cells were cultured in RPMI-1640 medium (Gibco, USA) and F12 medium (Gibco, USA) respectively supplemented with 10% fetal bovine serum (Gibco, USA). Both cell lines were incubated at 37°C in a humidified atmosphere containing 5% CO_2_. Chemical modified Stealth™ JMJD2B siRNA and scramble siRNA were purchased from Invitrogen. β-catenin siRNA was bought from GenePharma company (Shanghai, PR China). The sequences for JMJD2B and control siRNA were: JMJD2B siRNA, 5′-UCU CCA UCA CCU GCC UCA AGC ACA A-3′; control siRNA, 5′- CCU ACA UCC CGA UCG AUG AUG UUG A-3′. Lipofectamine 2000 (Invitrogen, USA) was used to transfect siRNAs into cells according to the protocol. PcDNA3.1-CagA plasmid was kindly provided by Yongliang Zhu (Zhejiang University, China) and it was described previously [35]. PcDNA3.1-CagA plasmid and pcDNA3.1 vector were transfected with X-treme GENE HP Transfection Reagent (Roche Diagnostics, Germany) according to the protocol. TCF/LEF-1 reporter (TOP-FLASH) and mutation vector (FOP-FLASH) were bought from Addgene (Addgene plasmid 12456 and 12457, Cambridge, MA).

### *H. pylori* strains and bacterial culture

*H. pylori* strains (NCTC 11637, 26695 and SS1) were kindly provided by Dr. Jianzhong Zhang (Chinese Disease Control and Prevention Center; Beijing, China). All strains were inoculated into Brucella broth containing 5% FBS under microaerobic conditions (5% O_2_, 10% CO_2_, and 85% N_2_) at 37°C. After 48h, *H. pylori* were collected and resuspended in phosphate buffered saline. Heat-killed *H. pylori* suspensions were prepared by incubating bacteria at 70°C for 10min followed by 95°C for 5 min.

### RNA extraction and quantitative real-time PCR (qRT-PCR)

Total RNA were extracted using Trizol reagent (Invitrogen, USA). After quantification, RNA was reverse transcribed into cDNA with RevertAid First Strand DNA Synthesis (RT) kit (Fermentas, Canada). Then, quantitative real-time PCR was performed with the SYBR Premix Ex Taq system (TaKaRa) using the Bio-Rad CFX96™ Real-Time PCR System (Bio-Rad). β-actin was used as an endogenous control. The primer sequences were shown in Table 4. Relative expression of the target genes were determined using the 2^−ΔΔCt^ method.

**Table 4:** primers for PCR

Genes	Primers
JMJD2B	5′-CCAGAGGCTTCCTTGCAGACAA-3′ (forward)
	5′-CCAAACTCCTGCCTCAGCCATT-3′ (reverse)
β-catenin	5′-GAAGGTGTGGCGACATATGCA-3′ (forward)
	5′-ATCCAAGGGGTTCTCCCTGGGC-3′ (reverse)
COX-2	5′-GCCCAGCACTTCACGCATCAG-3′ (forward)
	5′-AGACCAGGCACCAGACCAAAGACC-3′ (reverse)
β-actin	5′-AGTTGCGTTACACCCTTTCTTG-3′ (forward)
	5′-CACCTTCACCGTTCCAGTTTT-3′ (reverse)

### Western blot analysis

Total proteins were lysed with RIPA lysis buffer containing proteinase inhibitor, PMSF (Solarbio). Protein concentration was determined by the BCA reagent kit (Beyotime). Equal amounts of protein (80ug) were separated by 10% SDS-PAGE and transferred to PVDF membranes, which were blocked in 5% non-fat milk, and then incubated with the primary antibodies against JMJD2B (Bethyl Laboratories, Montgomery, USA), COX-2 (Cayman chemical, USA), β-catenin (Cell Signaling Technology, Billerica, USA) and β-actin (Santa Cruz Biotechnology, Santa Cruz, USA) at 4°C overnight. The membranes were then washed in TBS-T and incubated with anti-mouse or rabbit horseradish peroxidase-conjugated secondary antibodies and developed with the enhanced chemiluminescence method (ECL, Millpore). β-actin was a loading control.

### Coimmunoprecipitation (Co-IP)

Cells infected with *H. pylori* were lysed in RIPA lysis buffer. Extracts were clarified by centrifugation to collect the supernatant. The IP antibody aganist JMJD2B (Bethyl Laboratories, Montgomery, USA), p65 (ab7970, abcam, USA) and IgG (Santa Cruz Biotechnology) was added to the protein, and the mixture was gently shaken and incubated at 4°C for 5h. Then the solution was added with 20μl protein G PLUS-Agarose beads (Santa Cruz Biotechnology) at 4°C overnight. Immunoprecipitates were collected, washed, lysed, and boiled for Western blot analysis.

### Chromatin immunoprecipitation (ChIP)

The ChIP assay was performed in AGS cells using an immunoprecipitation assay kit (Millipore, USA) according to the protocol provided. Immunoprecipitated DNA sample was amplified using primers specific to the COX-2 promoter: 5′-GGCAAAGACTGCGAAGAAGA-3′ (Forward); 5′-ATTGCGTAAGCCCGGTGGG-3′ (Reverse) Quantitative real-time PCR was carried out using the Bio-Rad CFX96™ Real-Time PCR System (Bio-Rad). The amount of DNA coprecipitated with specific antibody was calculated in comparison to the total input DNA.

### Luciferase reporter assay

AGS and GES-1 cells were seeded in 24-well plates and JMJD2B promoter vector (−1022/+12), COX-2 promoter vector (−327/+19), pTOP-FLASH or pFOP-FLASH reporter plasmids (0.5 ug each) were separately transfected into the cells. After 48h, cells were lysed in passive lysis buffer and luciferase activity was measured by use of the Dual-Luciferase Reporter Assay System (Promega, Madison, WI, USA), and the target promoter-driven firefly luciferase activity was normalized to that of the Renilla control. Each experiment was performed in triplicate and repeated three times.

### Colony formation assay

AGS and GES-1 cells were infected with *H. pylori* or transfected with JMJD2B siRNA or control siRNA for corresponding time, and seeded into 6-well plates (300 cells/well) incubating for 10 days. Then, cells in the plates were fixed with methanol and stained with crystal violet, and the number of colonies with more than 50 cells was counted.

### Immunohistochemistry

Paraffin-embedded human gastric tissues underwent deparaffinization and antigen retrieval, then were incubated with primary antibodies against JMJD2B (1:100, Bethyl Laboratories, USA), COX-2 (1:100, Cayman chemical, USA) at 4°C overnight. Then slides were incubated with horseradish peroxidase-conjugated goat anti-rabbit secondary antibodies and developed with use of the DAB Kit (Gene Tech, shanghai, China). Slides were finally counterstained with hematoxylin.

### Patient specimens

Human gastritis specimens were collected from 52 patients undergoing gastroscopy. None of the patients had taken drugs such as antibiotics or anti-inflammatory drugs before the examination. Resected tissues from 32 patients with gastric cancer were obtained during surgery. None of the patients had received radiotherapy or chemotherapy before surgery. All the tissues from patients were obtained from Qilu Hospital, Shandong University (Jinan, PR China). The study was approved by the local ethics committee.

### Animal experiment

Specific-pathogen-free male C57BL/6J mice of 6-week-old (the Experimental Animal Center of Shandong University, China) were used in this study. We randomly divided 10 mice into 2 groups which were control group (n=5) and infection group (n=5). The infection group received 1 × 10^9^ colony-forming units of *H. pylori* SS1 by intragastric gavage for 3 times after 12h fasting every other day. The control group received 1mL PBS each time in the same way. After eight weeks, mice were sacrificed and gastric mucosal samples were collected for JMJD2B and COX-2 detection. All animal experimental protocols were approved by the local Ethics Committee of Shandong University.

### Statistical analysis

Comparisons between different groups were analyzed by Student's *t*-test. The correlation between JMJD2B and COX-2 expression was evaluated with the χ^2^ test. P< 0.05 was considered statistically significant.
